# Improved Simulation of Electrodiffusion in the Node of Ranvier by Mesh Adaptation

**DOI:** 10.1371/journal.pone.0161318

**Published:** 2016-08-22

**Authors:** Ibrahima Dione, Jean Deteix, Thomas Briffard, Eric Chamberland, Nicolas Doyon

**Affiliations:** 1 Département de mathématiques et de statistique/Groupe Interdisciplinaire de Recherche en Éléments Finis (GIREF), Université Laval, Québec, Québec, Canada; 2 Neurosciences cellulaires et moléculaires/Institut universitaire en santé mentale de Québec (CRIUSMQ), Université Laval, Québec, Québec, Canada; McGill University Department of Physiology, CANADA

## Abstract

In neural structures with complex geometries, numerical resolution of the Poisson-Nernst-Planck (PNP) equations is necessary to accurately model electrodiffusion. This formalism allows one to describe ionic concentrations and the electric field (even away from the membrane) with arbitrary spatial and temporal resolution which is impossible to achieve with models relying on cable theory. However, solving the PNP equations on complex geometries involves handling intricate numerical difficulties related either to the spatial discretization, temporal discretization or the resolution of the linearized systems, often requiring large computational resources which have limited the use of this approach. In the present paper, we investigate the best ways to use the finite elements method (FEM) to solve the PNP equations on domains with discontinuous properties (such as occur at the membrane-cytoplasm interface). **1)** Using a simple 2D geometry to allow comparison with analytical solution, we show that mesh adaptation is a very (if not the most) efficient way to obtain accurate solutions while limiting the computational efforts, **2)** We use mesh adaptation in a 3D model of a node of Ranvier to reveal details of the solution which are nearly impossible to resolve with other modelling techniques. For instance, we exhibit a non linear distribution of the electric potential within the membrane due to the non uniform width of the myelin and investigate its impact on the spatial profile of the electric field in the Debye layer.

## Introduction

Since the pioneer work of Hodgkin and Huxley [1], mathematical modelling of the electric activity of neurons has become an important tool to investigate the nervous system. While most models have relied on the cable theory formalism based on the strong analogy between neurons and electric circuits, limits of this formalism include its incapacity to account for fluctuations of ionic concentrations or to describe the electric field beyond the membrane. Extensions of the cable theory formalism have been developed [2, 3], notably in the NEURON software environment, in order to describe changes in ionic concentration as well as the impact of intracellular chemical reactions [4]. However, these extensions still fail to accurately describe the electric field beyond the membrane or the distribution of ionic concentrations in the Debye layer [5].

Solving the Poisson-Nernst-Planck (PNP) partial differential equations is a promising approach to overcome these limitations and model the evolution of ionic concentrations and of the electric field in neural structures such as axons, nodes of Ranvier, dendritic spines or the synaptic cleft ([5–9]). This strategy, not relying on oversimplifying assumptions such as the charge difference between intra and extracellular media being localized at the membrane, offers a potential spatial resolution in the nanometer range [7].

The FEM requires a spatial partition of the computational domain with a mesh of either tetrahedral or hexahedral elements and looks for a piecewise polynomial function on each of these elements which is the best approximation of the physical solution. In general, this renders the FEM unable to describe phenomena with spatial extents much smaller than the size of a mesh element which can potentially lead to exploding computational costs. For instance, the description of a volume of a cubic micron with an uniform resolution of ten nanometers would require a mesh of roughly one million elements. Modelling the evolution of four ionic concentrations as well as of the electric field on such a mesh using piecewise linear polynomials would imply having to solve 16 millions equations at each time step leading to either unreasonably slow or plainly infeasible calculations.

A clever way to reduce the computational cost without compromising on solution quality is to concentrate the mesh elements where the solution exhibits abrupt variations while tolerating a coarser mesh elsewhere. Such meshes, as the one used in [5], are created and tailored (adjusted) based on the knowledge of the behaviour of the solution, we will call them “tailored meshes”. It is also possible to rely on the mesh adaptation method which is based on error estimates derived from preliminary solutions [10–12]. Mesh adaptation is successfully applied in many industrial problems. While its first goal is to increase accuracy of the simulation for a given numerical effort, one can also view this approach as an automatic and objective method of constructing meshes based on intrinsic properties of the simulated quantities. As opposed to a “tailored mesh”, mesh adaptation doesn’t rely on the user a priori beliefs about the solution properties and remain easily applicable to complex geometries.

In neural structures, ionic concentrations as well as the electric potential are almost uniform over large parts of both the intracellular and extracellular space while experiencing steep variations in the vicinity of the membrane or in regions of high current density like the node of Ranvier or synapses. In the absence of transmembrane flux, variations in ionic concentrations and membrane potential are localized in the so called Debye layer [13] with a characteristic length of about 1 nm. These observations make the use of adapted meshes particularly promising in the field of computational neuroscience.

In the present paper, we first provide a detailed discussion on how to best apply the FEM to the resolution of the PNP equations in the field of computational neuroscience bearing in mind to make the use of our method by other research groups as straightforward as possible. Then, using a simple 2D geometry without transmembrane currents, we show that some kind of mesh adaptation (by an automatic process or by a “manual” positioning of the nodes) is *necessary* to obtain accurate solutions as resolution on an **uniform** mesh leads to sizeable numerical errors and for insufficiently refined meshes failed to converge to the exact value of the membrane potential obtained analytically. In the absence of transmembrane current, simulations based on automatic mesh adaptation presented here concentrate mesh nodes in the first nanometer from the membrane (in the Debye layer) exactly where the variations of the solution are expected to occur. We also compared the quality of solutions obtained on tailored meshes in which the node density decreased geometrically as a function of distance from the membrane (similar to the meshes in [5]). While both strategies led to important concentration of mesh nodes in the Debye layer, for a similar number of mesh nodes, the simulation on adapted mesh led to important gain in accuracy (in certain cases we observed a reduction by a factor of almost ten of the numerical error).

We finally model the generation of an action potential in a 3 dimensional model of a node of Ranvier. This establishes the feasibility of applying this approach to complex geometries thus to physiologically relevant investigations of structures such as dendritic spines, synaptic clefts or multiple nodes of Ranvier in nerve bundles [14]. Beyond that, our simulations reveal details of the solutions that cannot be captured by other approaches. For instance, we show that in the nodal region in the vicinity of the membrane, the electric potential can deviate by as much as 0.5 mV from neutrality (larger than predicted by models with uniform membrane width [5]) and that the electric potential varies in a highly non linear way in the membrane at the junction of the myelinated and non myelinated region. To our knowledge, our approach is the first to simultaneously describe the nanometric Debye layer and the complex geometry of a node of Ranvier.

It is our belief that those two tests provide a good illustration that simulations based on adapted meshes are the most efficient from a numerical point of view. Moreover, it can capture unsuspected minute details of the solution that cannot be detected without a priori knowledge. It is possible to argue that the use of adapted meshes could be neglected for oversimplified geometries as “manual” positioning of the nodes can also lead to good results. However, even in those simple cases mesh adaptation reduces the numerical efforts leading to shorter simulation times for a fixed accuracy level. More importantly, adapted meshes can be used on complex geometries for which “tailored meshes” are difficult or plainly impossible to construct.

## Modelling methodology

### Electro-diffusion theory and model

The electrodiffusion model solves the system of partial differential equations including the Nernst-Planck equation

$$\frac{\partial c_{k}}{\partial t}=\nabla \cdot [D_{k}\left(\nabla c_{k}+\frac{c_{k}}{\alpha_{k}}\nabla V\right)],\;\text{in}\;\Omega ,\;\;\;k=1,\cdots ,n^{*},$$ (1)

which describes the fluxes of n* ionic species subjected to electro-diffusion, as well as the Poisson equation

$$-\nabla \cdot \left(\varepsilon \nabla V\right)=F\sum_{k=1}^{n^{*}}z_{k}c_{k},\;\text{in}\;\Omega ,$$ (2)

which relates the electric potential *V* to the distribution of the electric charges. Here *c*_*k*_, *D*_*k*_ and *z*_*k*_ are the concentration, diffusion coefficient and valence of the ionic specie *k* respectively. The coefficient *α*_*k*_ is defined by *α*_*k*_ = *RT*/*Fz*_*k*_, where *R* is the perfect gas constant, *F* the Faraday constant, *T* the absolute temperature and *ε* is the dielectric constant of the medium *Ω*. If we define **F**_*k*_, by

$$F_{k}=-D_{k}\left(\nabla c_{k}+\frac{c_{k}}{\alpha_{k}}\nabla V\right),$$ (3)

as the flux of ionic specie *k*, then Eq (1) can be rewritten as the following continuity equation

$$\frac{\partial c_{k}}{\partial t}+\nabla \cdot F_{k}=0,\;\text{in}\;\Omega ,\;\;\;k=1,\cdots ,n^{*}.$$ (4)

We solved Eqs (1) and (2) on a computational domain *Ω* composed of three parts: the intracellular space, the membrane and the extracellular space. The diffusion coefficients of ionic species in the intra and extracellular spaces are taken to be the diffusion coefficients in water (see Table 1 and [15]) while these coefficients are set to zero on the membrane where the ionic concentrations themselves are also taken to be zero. The dielectric permittivity constant in both the intracellular and extracellular spaces is taken to be the dielectric permittivity constant of water *ε*_0_ *ε*_*w*_ where *ε*_0_ is the dielectric permittivity constant of vacuum and *ε*_*w*_ ≈ 80 is the relative dielectric permittivity of water [16] (see Table 1) while the dielectric permittivity of the membrane is given by *ε*_0_ *ε*_mem_ where *ε*_mem_ is the relative dielectric permittivity of the membrane which value will be discussed below.

**Table 1:** Electrodiffusion parameters.

*R*	8.31454 J ⋅ mole^−1^ ⋅ K^−1^	Perfect gas constant
*F*	96485 C ⋅ mole^−1^	Faraday constant
*T*	279.450 K	Absolute temperature
*ε*_0_	8.88541 ⋅ *e*^−12^ C ⋅ m^−1^ ⋅ V^−1^	Vacuum electric permittivity
*ε*_*w*_	80	Water relative dielectric permittivity
*ε*_mem_	40	Membrane relative dielectric permittivity
[K^+^]_*i*_	155mM	Initial intracellular K^+^ concentration
[Na^+^]_*i*_	12mM	Initial intracellular Na^+^ concentration
[A^−^]_*i*_	167.02mM	Initial intracellular anion concentration
[K^+^]_*o*_	4mM	Initial extracellular K^+^ concentration
[Na^+^]_*o*_	145mM	Initial extracellular Na^+^ concentration
[A^−^]_*o*_	149mM	Initial extracellular anion concentration
*D*_*K*_	1.96*μ*m^2^ ⋅ ms^−1^	K^+^ diffusion coefficient
*D*_*Na*_	1.33*μ*m^2^ ⋅ ms^−1^	Na^+^ diffusion coefficient
*D*_*A*_	2.00*μ*m^2^ ⋅ ms^−1^	Anion diffusion coefficient.

While Eqs (1) and (2) could be used to describe an arbitrarily large number of ionic concentrations, we limited ourselves to a minimal set of cations K^+^, Na^+^ and a generic anion species A^−^. The first two are needed for the description of an action potential, whereas the last one is needed to obtain realistic net charge, osmolarity and cytosol resistivity [6]. Initial concentrations in the intracellular and extracellular domains are given in Table 1. For both concentrations and electric potential, we impose fixed Dirichlet conditions on the outermost boundary of the extracellular space and denote this boundary by $\Gamma_{ex}^{t}$. For ionic concentrations, we apply for each ionic species *k* = 1, ⋯, n*, the non-homogeneous boundary condition

$$c_{k}=c_{k}^{0},\;\text{on}\;\Gamma_{ex}^{t},$$ (5)

where $c_{k}^{0}$ take the initial value of the extracellular concentration of ionic specie *k*, whereas the voltage obeys to the following homogeneous Dirichlet condition

$$V=0,\;\text{on}\;\Gamma_{ex}^{t}.$$ (6)

On the outermost boundaries of the intracellular space denoted by $\Gamma_{ex}^{\ell }$ and $\Gamma_{ex}^{r}$, we apply the following homogeneous Neumann boundary conditions for each ionic species *k* = 1, ⋯, n* and for the potential

$$F_{k}\cdot n=0,\;\text{on}\;\Gamma_{ex}^{r}\cup \Gamma_{ex}^{\ell },$$ (7)

$$\nabla V\cdot n=0,\;\text{on}\;\Gamma_{ex}^{r}\cup \Gamma_{ex}^{\ell }.$$ (8)

The boundary of the domain Ω is then given by $\partial \Omega ≔{\bar{\Gamma }}_{ex}^{t}\cup {\bar{\Gamma }}_{ex}^{r}\cup {\bar{\Gamma }}_{ex}^{\ell }$. Moreover on the electrolyte-membrane interfaces, additional homogeneous Neumann conditions are applied for concentrations, except around the node of Ranvier where non zero flux is applied for concentrations. Denoting by $\Gamma_{in}^{n}$ the two cylindrical surfaces defining the intracellular-membrane and the extracellular-membrane interfaces at the node of Ranvier, we apply for every ionic specie

$$F_{k}\cdot n=f_{k},\text{on}\;\Gamma_{in}^{n},$$ (9)

where functions *f*_*k*_ are computed using the conductance of voltage-gated channels. The details of flux computation and the dynamics of the gating variables follow the standard Hodgkin-Huxley scheme and are given in the supplemental methods (S1 Methods). The vector **n** is the unit outer normal at the associated boundaries.

### Weak formulation of the Electro-diffusion model

Multiplying Eq (4), for each ionic species *k* = 1, ⋯, n*, by proper test functions *ψ* and integrating over the domain Ω, we obtain

$$\int_{\Omega }\frac{\partial c_{k}}{\partial t}\;\psi \;dx+\int_{\Omega }\nabla \cdot F_{k}\;\psi \;dx=0.$$ (10)

For the electric potential, we multiply Eq (2) by the test function *φ* and obtain

$$-F\sum_{k=1}^{n^{*}}\int_{\Omega }z_{k}c_{k}\varphi \;dx-\int_{\Omega }\nabla \cdot \left(\varepsilon \nabla V\right)\varphi \;dx=0.$$ (11)

Applying integration by parts on Eqs (10) and (11) yields

$$\begin{array}{lll} \int_{\Omega }\frac{\partial c_{k}}{\partial t}\psi dx+\int_{\Omega }D_{k}\nabla c_{k}\cdot \nabla \psi dx & + & \int_{\Omega }{\tilde{D}}_{k}c_{k}\nabla V\cdot \nabla \psi dx=\\ & - & \int_{\partial \Omega \cup \Gamma_{in}^{n}}\left(F_{k}\cdot n\right)\psi ds,\\ -F\overset{n^{*}}{\underset{k=1}{\sum }}\underset{\Omega }{\int }z_{k}c_{k}\varphi dx+\underset{\Omega }{\int }\varepsilon \nabla V\cdot \nabla \varphi dx & = & \int_{\partial \Omega }\left(\varepsilon \nabla V\cdot n\right)\varphi ds, \end{array}$$

where ${\tilde{D}}_{k}≔\frac{D_{k}}{\alpha_{k}}$. Taking into account Dirichlet boundary conditions Eqs (5) and (6), we denote by ℂ and V functional spaces of concentrations and electric potential, respectively

$$C=V=\left\{\varphi \in H^{1}\left(\Omega \right),\;\varphi =0\;\text{on}\;\Gamma_{ex}^{t}\right\}.$$ (12)

Reorganizing the previous system, we obtain the following so called weak formulation of the PNP equations: Find *c*_*k*_ ∈ *H*^1^(Ω) with $c_{k}-c_{k}^{0}\in \mathbb{C}$, *k* = 1, ⋯, n*, and V∈V such that

$$\int_{\Omega }\frac{\partial c_{k}}{\partial t}\psi \;dx+\int_{\Omega }D_{k}\nabla c_{k}\cdot \nabla \psi \;dx+\int_{\Omega }{\tilde{D}}_{k}c_{k}\nabla V\cdot \nabla \psi \;dx=-\int_{\Gamma_{in}^{n}}f_{k}\;\psi \;ds,$$ (13)

$$\begin{array}{lll} & & \forall \psi \in \mathbb{C},\\ -F\overset{n^{*}}{\underset{k=1}{\sum }}\int_{\Omega }z_{k}c_{k}\varphi dx+\int_{\Omega }\varepsilon \nabla V\cdot \nabla \varphi dx & = & 0,\forall \varphi \in V. \end{array}$$ (14)

### Finite element approximations

The finite element treatment of the PNP equations first requires a spatial discretization of Eqs (13) and (14) by representing unknown functions *c*_*k*_ and *V* as well as test functions *ψ* and *φ* by piecewise polynomials on the mesh $T_{\eta }≔\{⊤\}$ of simplicial ⊤ partitioning the domain Ω. In fact we consider the following finite approximation spaces ${\mathbb{C}}_{\eta }$ and $V_{\eta }$ of piecewise polynomials of degree *q* and *p* defined relatively to the mesh $T_{\eta }$:

$$H_{\eta }^{k^{*}}:=\left\{\psi_{\eta }\in C^{0}\left(\bar{\Omega }\right):\;\psi_{\eta }\in P_{k^{*}}\left(⊤\right)\right\},$$

$$C_{\eta }:=\left\{\psi_{\eta }\in H_{\eta }^{q},\;\psi_{\eta }=0\;\text{on}\;\Gamma_{ex}^{t}\right\},$$ (15)

$$V_{\eta }:=\left\{\varphi_{\eta }\in H_{\eta }^{p},\;\varphi_{\eta }=0\;\text{on}\;\Gamma_{ex}^{t}\right\},$$ (16)

where $P_{k}{}_{*}\left(⊤\right)$ is the space of polynomials of degree k* on ⊤. The discrete weak formulation in space of Eqs (13) and (14) is then given by: Find approximated solutions $c_{k,\eta }\in {ℍ}_{\eta }^{q}$ with $c_{k,\eta }-c_{k,\eta }^{0}\in {\mathbb{C}}_{\eta },k=1,\ldots ,n^{*}$ and $V_{\eta }\in V_{\eta }$ such that

$$\begin{array}{c} \underset{\Omega }{\int }\frac{\partial c_{k,\eta }}{\partial t}\psi_{\eta }dx+\underset{\Omega }{\int }D_{k}\nabla c_{k,\eta }\cdot \nabla \psi_{\eta }dx+\underset{\Omega }{\int }{\tilde{D}}_{k}c_{k,\eta }\nabla V_{\eta }\cdot \nabla \psi_{\eta }dx\\ =-\underset{\Gamma_{in}^{n}}{\int }f_{k}\psi_{\eta }ds,\forall \psi_{\eta }\in {\mathbb{C}}_{\eta }, \end{array}$$ (17)

$$-F\sum_{k=1}^{n^{*}}\int_{\Omega }z_{k}c_{k,\eta }\varphi_{\eta }\;dx+\int_{\Omega }\varepsilon \nabla V_{\eta }\cdot \nabla \varphi_{\eta }\;dx=0,\;\forall \;\varphi_{\eta }\;\in \;V_{\eta },$$ (18)

where $c_{k,\eta }^{0}$ is the interpolation of the initial value function $c_{k}^{0}$ onto ${ℍ}_{\eta }^{q}$.

For the discretization in time, we consider a second order backward difference formula (BDF2) (also known as Gear time stepping scheme, it is an implicit multistep marching scheme) [17]. This numerical procedure, for solving an ordinary differential equation of the type

$$\frac{\partial y}{\partial t}=F\left(t,y\right),$$ (19)

can be exemplified as follows: To obtain the approximation *y*_*r*+1_ of the solution *y* at time step *t*_*r*+1_ = *t*_*r*_ + Δ*t*_*r*_, solve the following equation

$$\frac{3}{2}y_{r+1}=2y_{r}-\frac{1}{2}y_{r-1}+\Delta t_{r}F\left(t_{r+1},y_{r+1}\right)$$ (20)

which depends on values that are yet unknown. This implicit scheme is of second order accuracy, meaning that the error scales with $O(\Delta t_{r}^{2})$. Applying it to the discrete weak formulation in space Eqs (17) and (18), we seek approximation of solutions *c*_*k*, *η*_ and *V*_*η*_ as follows: Given functions $c_{k,\eta }^{r-1}$, $c_{k,\eta }^{r}$, $V_{\eta }^{r-1}$ and $V_{\eta }^{r}$, for times *t*_*r*−1_ and *t*_*r*_ respectively, find $c_{k,\eta }^{r+1}\in {ℍ}_{\eta }^{q}$ with $c_{k,\eta }^{r+1}-c_{k,\eta }^{0}\in {\mathbb{C}}_{\eta }$ and $V_{\eta }^{r+1}\in V_{\eta }$ such that

$$\int_{\Omega }\frac{1}{\Delta t_{r}}\left(\frac{3}{2}c_{k,\eta }^{r+1}-2c_{k,\eta }^{r}+\frac{1}{2}c_{k,\eta }^{r-1}\right)\psi_{\eta }\;dx+\int_{\Omega }D_{k}\nabla c_{k,\eta }^{r+1}\cdot \nabla \psi_{\eta }\;dx+\int_{\Omega }{\tilde{D}}_{k}c_{k,\eta }^{r+1}\nabla V_{\eta }^{r+1}\cdot \nabla \psi_{\eta }\;dx=-\int_{\Gamma_{in}^{n}}f_{k}\;\psi_{\eta }\;ds,\forall \;\psi_{\eta }\;\in C_{\eta },$$ (21)

$$-F\sum_{k=1}^{n^{*}}\int_{\Omega }z_{k}c_{k,\eta }^{r+1}\varphi_{\eta }\;dx+\int_{\Omega }\varepsilon \nabla V_{\eta }^{r+1}\cdot \nabla \varphi_{\eta }\;dx=0,\;\forall \;\;\varphi_{\eta }\;\in \;V_{\eta }.$$ (22)

Expressing trial functions $c_{k,\eta }^{r+1}(x)$ and $V_{\eta }^{r+1}(x)$ as weighted sum of basis functions $\{\psi_{i}{\}}_{i=1}^{N_{q}}$ and $\{\varphi_{i}{\}}_{i=1}^{N_{p}}$ for ${\mathbb{C}}_{\eta }$ and $V_{\eta }$ respectively,

$$c_{k,\eta }^{r+1}\left(x\right):=\sum_{i=1}^{N_{q}}c_{k,i}^{r+1}\psi_{i}\left(x\right)\;,\;k=1,...,n^{*}\;\;V_{\eta }^{r+1}\left(x\right):=\sum_{i=1}^{N_{p}}V_{i}^{r+1}\varphi_{i}\left(x\right),$$ (23)

and taking test functions as *ψ*_*η*_ = *ψ*_*j*_, *j* = 1, …, *N*_*q*_ and *φ*_*η*_ = *φ*_*j*_, *j* = 1, …, *N*_*p*_, the system Eqs (21) and (22) is rewritten as an algebraic system of (n* × *N*_*q*_)+*N*_*p*_ equations

$$\left(\begin{array}{cc} A_{11} & A_{12}\left(c^{r+1}\right)\\ A_{21} & A_{22} \end{array}\right)\left(\begin{array}{c} c^{r+1}\\ V^{r+1} \end{array}\right)=\left(\begin{array}{c} F_{1}\left(c^{r+1},V^{r+1}\right)\\ F_{2}\left(c^{r+1},V^{r+1}\right) \end{array}\right)$$

where $c^{r+1}=(c_{1}^{r+1},...,c_{n^{*}}^{r+1})\in {\mathbb{R}}^{n^{*}\times N_{q}}$ and $V^{r+1}\in {\mathbb{R}}^{N_{p}}$ are vectors associated with the coefficients $c_{k,i}^{r+1}$ and $V_{i}^{r+1}$ in Eq (23). The coupling term in Eq (21) (third term on the left hand side) makes this an algebraic nonlinear system of generic form

$$A\left(U^{r+1}\right)U^{r+1}=F\left(U^{r+1}\right).$$

To solve such problem at time step *r*+1, the Newton-Raphson method is used. Starting with *U*_1_ = *U*^*r*^ (the solution at the preceding time step), a first order approximation of the linearized system is solved iteratively,

$$\begin{array}{ccc} K_{U}\delta_{U} & = & F\left(U_{s}\right)-A\left(U_{s}\right)U_{s}\\ U_{s+1} & = & \delta_{U}+U_{s} \end{array}$$ (24)

and *U*^*r*+1^ is obtained at the end of this process (the loop is stopped when the correction *δ*_*U*_ is small enough). In our case, the main difficulty in the definition of the tangent matrix $K_{U}$, arises from the coupling term. Introducing *δ*_*c*_, *δ*_*V*_ and neglecting the second order terms, we get

$$\int_{\Omega }{\tilde{D}}_{k}c_{s+1}^{k}\nabla V_{s+1}\cdot \nabla \psi_{\eta }\;dx\approx \int_{\Omega }{\tilde{D}}_{k}c_{s}^{k}\nabla V_{s}\cdot \nabla \psi_{\eta }\;dx+\int_{\Omega }{\tilde{D}}_{k}\delta_{c}\nabla V_{s}\cdot \nabla \psi_{\eta }\;dx\;+\int_{\Omega }{\tilde{D}}_{k}c_{s}^{k}\nabla \delta_{V}\cdot \nabla \psi_{\eta }\;dx.$$

**Algorithm 1**: Finite element resolution

1. Given a mesh, the initial time *t*_0_, $c_{1}^{0}$, …,$c_{n^{*}}^{0}$ and **V**^0^ the initial conditions. Put *r* = 0;

2. **While** [final time is not reached]

 • Compute a time step length Δ*t*_*r*_, *t*_*r*+1_ = *t*_*r*_+Δ*t*_*r*_

 • Compute the flux *f*_*k*_ by solving a system of ordinary differential equations (see S1 Methods)

 • Put $V_{1}^{k}=V_{k}^{r},\;\;k=1,...,n^{*}$, **V**_1_ = *V*^*r*^ and *s* = 1

 • **While** [desired tolerances on $\delta_{c}^{k}$ and *δ*_*V*_ are not reached]

  - Construct the matrices ${\hat{K}}_{cc}^{k}=\gamma M_{c}+D_{c}^{k}+D_{cc}^{k},\;\;D_{cv}^{k},\;\;S^{k}\;\;k=1,...,n^{*}$, **D**_*v*_

  - Construct the vectors $r_{c}^{k}=F_{c}^{k}-M_{c}{\tilde{c}}_{n}^{k}-{\hat{K}}_{cc}^{k}c_{s}^{k},r_{v}=-\sum_{k=1}^{n*}S^{k}c_{s}^{k}-D_{v}V_{m}$

  - Solve the linear system

    $\begin{array}{lll} {\hat{K}}_{cc}^{k}\delta_{c}^{k}+D_{cv}^{k}\delta_{v} & = & r_{c}^{k},\;k=1,\ldots ,n*\\ \sum_{k=1}^{n*}S^{k}\delta_{c}^{k}+D_{v}\delta_{V} & = & r_{v}, \end{array}$

  - Put $\;c_{s+1}^{k}=\delta_{c}^{k}+c_{s}^{k},\;k=1,...,n^{*},\;c_{s+1}=\delta_{v}+c_{s}$

  - Set *s* = *s* + 1

 • **End while**

 • Compute the approximation at time *t*_*r*+1_

    $c_{k}^{r+1}=c_{s+1}^{k},\;k=1,...,n^{*},\;V^{r+1}=V_{s+1}$

3. **End while**

Using this expression in Eqs (21) and (22) and the corrections *δ*_*c*_, *δ*_*V*_, the system Eq (24) is described by

$$\left(\gamma M_{c}+D_{c}^{k}+D_{cc}^{k}\right)\delta_{c}^{k}+D_{cv}^{k}\delta_{V}=F_{c}^{k}-M_{c}{\tilde{c}}_{n}^{k}-\left(\gamma M_{c}+D_{c}^{k}+D_{cc}^{k}\right)c_{s}^{k},$$ (25)

$$\sum_{k=1}^{n^{*}}S^{k}\delta_{c}^{k}+D_{v}\delta_{V}=-\sum_{k=1}^{n^{*}}S^{k}c_{s}^{k}-D_{v}V_{s},$$ (26)

$$c_{s+1}^{k}=\delta_{c}^{k}+c_{s}^{k},\;\;k=1,\cdots ,n^{*}\;V_{s+1}=\delta_{V}+V_{s}$$ (27)

where, at time step *r*+1, we take as a starting state: $c_{1}^{k}=c_{k}^{r}\;\;k=1,...,n^{*}$ and **V**_1_ = **V**^*r*^, the matrices and vectors are defined respectively by

$$\begin{array}{cccccc} {\left(M_{c}\right)}_{ij} & := & \int_{\Omega }\psi_{i}\psi_{j}\;dx, & {\left(D_{c}^{k}\right)}_{ij} & := & \int_{\Omega }D_{k}\nabla \psi_{i}\cdot \nabla \psi_{j}\;dx,\\ {\left(D_{cc}^{k}\right)}_{ij} & := & \int_{\Omega }{\tilde{D}}_{k}\psi_{j}\sum_{\ell =1}^{N_{p}}{\left(V_{s}\right)}_{\ell }\nabla \varphi_{\ell }\cdot \nabla \psi_{i}\;dx, & {\left(F_{c}^{k}\right)}_{j} & := & -\int_{\Gamma_{in}^{n}}f_{k}\;\psi_{j}\;ds,\\ {\left(D_{cv}^{k}\right)}_{ij} & := & \int_{\Omega }{\tilde{D}}_{k}\sum_{\ell =1}^{N_{q}}{\left(c_{s}^{k}\right)}_{\ell }\psi_{\ell }\nabla \varphi_{j}\cdot \nabla \psi_{i}\;dx, & {\tilde{c}}_{n}^{k} & := & -\beta c_{k}^{n}-\alpha c_{k}^{r-1}\\ S_{ij}^{k} & := & -F\int_{\Omega }z_{k}\psi_{i}\varphi_{j}\;dx, & {\left(D_{v}\right)}_{ij} & := & \int_{\Omega }\varepsilon \nabla \varphi_{i}\cdot \nabla \varphi_{j}\;dx \end{array}$$

$$\alpha =\frac{1}{2\Delta t_{r}}\;\beta =-\frac{2}{\Delta t_{r}}\;\gamma =\frac{3}{2\Delta t_{r}}$$

*α*, *β* and *γ* are the coefficients of the finite difference Eq (20). The complete sequence of approximation is presented in Algorithm 1.

It is possible to have the length of the time step Δ*t*_*r*_ changed as the calculation proceeds according to the dynamics of the system to speed up the simulation. Here we used the fact that the number of iterations for the resolution of one time step increases with Δ*t*_*r*_ to reach a target range [*N*_min_, *N*_max_] of number of iterations for each time step. If *N*_it_ is the number of iterations at *t*_*r*_, then for *t*_*r*+1_ we define

$$\Delta t_{r}=\left\{\begin{array}{cc} \left(1+f_{\text{min}}\right)\Delta t_{r-1} & N_{\text{it}}<N_{\text{min}},\\ \Delta t_{r-1} & N_{\text{min}}\leq N_{\text{it}}\leq N_{\text{max}},\\ \left(1-f_{\text{max}}\right)\Delta t_{r-1} & N_{it}>N_{\text{max}}. \end{array}\right.$$

For the discretization of the system Eqs (13) and (14) we chose quadratic interpolation $P_{2}(⊤)$ for all spaces, therefore k* = *p* = *q* = 2 and ${\mathbb{C}}_{\eta }=V_{\eta }$. Although the use of linear interpolations (i.e. $P_{1}(⊤)$) could be viewed as less demanding (since it leads to smaller algebraic system), the higher degree of precision of quadratic interpolation makes it possible to use coarser meshes for the same precision.

The use of explicit time schemes for the system of Eqs (17) and (18) was excluded as it leads to conditional stability of the method and the Courant–Friedrichs–Lewy (CFL) condition [18] needed to insure stability would impose very small time steps. A simpler implicit time marching scheme could be used, for instance taking $\alpha =0,\;\;\beta =\frac{-1}{\Delta t_{r}},\;\;\gamma =\frac{-1}{\Delta t_{r}}$ corresponds to the backward Euler scheme (BDF1). Once again, even if this could be viewed as a reduction of computation, the result would be opposite since the diminution in precision imposes the use of smaller time steps therefore an increase in the total number of time steps.

In the same manner, simplifications in the construction of the matrix involved in the left hand side of Eq (25) could be seen as reducing the assemblage, therefore decreasing computation time. However, this would lead to a degradation in the convergence rate of the iterative method thus to an increase in non linear iterations and overall calculation time. One such simplification could be to neglect the matrix contribution $D_{cc}^{k}$, still giving a converging method. Neglecting both terms of the linearisation ($D_{cc}^{k}$ and $D_{cv}^{k}$) produces a diverging method since a large part of the information related to the coupling is ignored.

### Mesh adaptation

**Fig 1:**
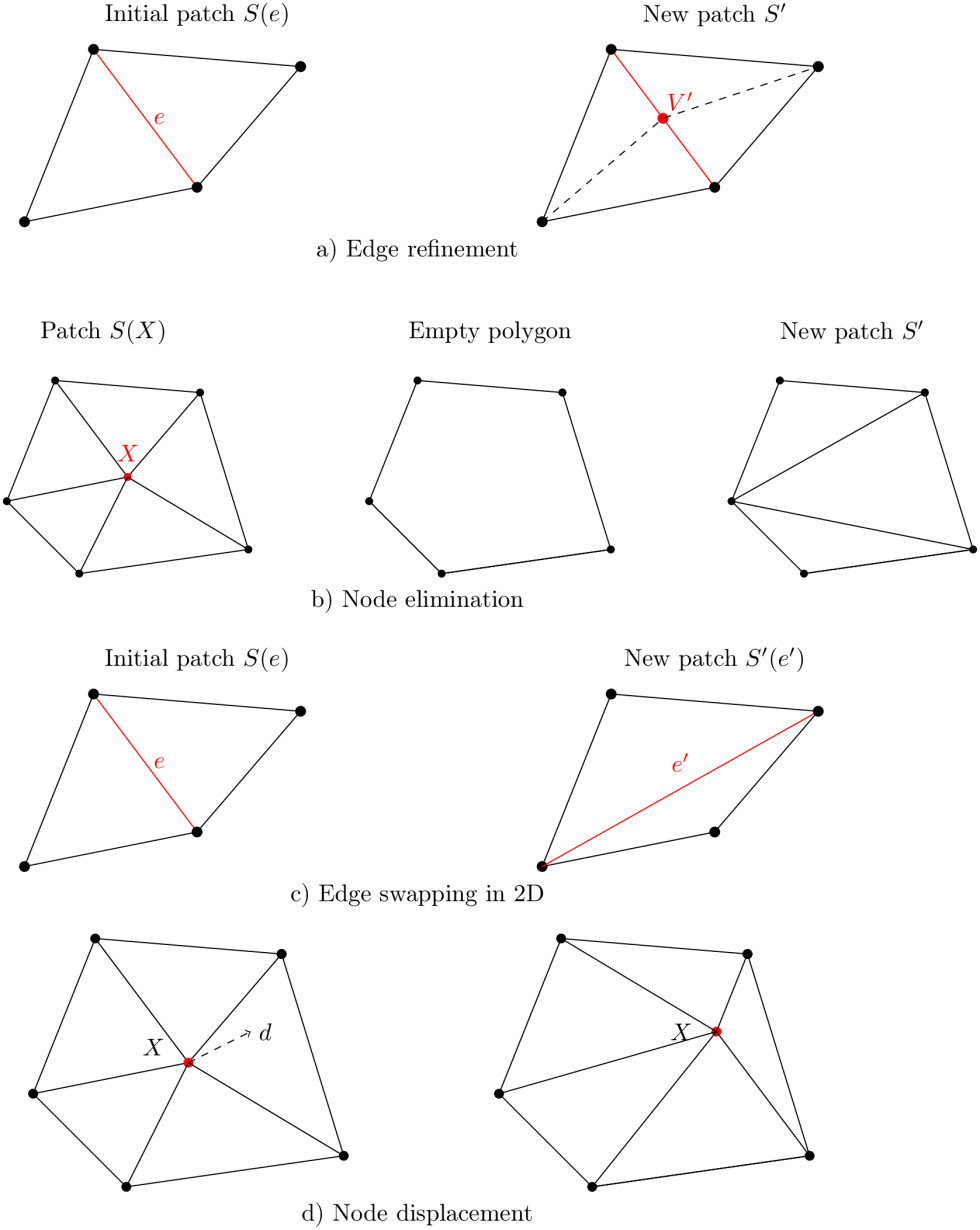
**a)** Edge refinement. **b)** Node elimination. **c)** Edge swapping in 2D. **d)** Node displacement.

Electric potential in neural structures is characterized by localized and fast transient behaviour. In such cases the accuracy of the numerical approximations often deteriorates due to phenomena such as: local singularities (like those arising from re-entrant corners of domains), excessively small zones of heterogeneous material, presence of boundary layers or sharp moving fronts. An obvious strategy to improve the quality of the solution is to refine the resolution grid but in situations where different spatial scales are involved, refining in a global manner will lead to an excessive work load. Mesh adaptation improves the quality of FEM solutions thanks to local operations such as edge refinement (Fig 1A), node elimination (Fig 1B), edge swapping (Fig 1C) and node displacement (Fig 1D).

We will briefly detail the main ideas behind our hierarchical error estimator and our adaptive remeshing strategy and we refer to [11, 12] for more details. Let $T_{\eta }$ denote a triangulation of the domain Ω and ⊤ its elements. Let *u* be the exact solution, usually unknown and suppose that we have computed a finite element approximation of degree *q* of the solution denoted $u_{\eta }^{\left(q\right)}$. We now make the assumption that we can build, starting from $u_{\eta }^{\left(q\right)}$, a new approximation ${\hat{u}}_{\eta }^{\left(q+1\right)}$ of degree *q* + 1 that is at least slightly more accurate in the sense that

$$\|u-{\hat{u}}_{\eta }^{\left(q+1\right)}\|\leq \beta \|u-u_{\eta }^{\left(q\right)}\|$$ (28)

with *β* < 1. The construction of ${\hat{u}}_{\eta }^{\left(q+1\right)}$ is therefore crucial and will be described later on. For the moment, we assume its existence and the triangle inequality gives

$$\|u-u_{\eta }^{\left(q\right)}\|\leq \|u-{\hat{u}}_{\eta }^{\left(q+1\right)}\left\|+\right\|{\hat{u}}_{\eta }^{\left(q+1\right)}-u_{\eta }^{\left(q\right)}\|\leq \beta \|u-u_{\eta }^{\left(q\right)}\left\|+\right\|{\hat{u}}_{\eta }^{\left(q+1\right)}-u_{\eta }^{\left(q\right)}\|$$

thus

$$\|u-u_{\eta }^{\left(q\right)}\|\leq \frac{1}{1-\beta }\left\|{\hat{u}}_{\eta }^{\left(q+1\right)}-u_{\eta }^{\left(q\right)}\right\|$$ (29)

and the error can then be controlled by the right-hand side in some appropriate norm (*L*^2^ or *H*^1^-norm for instance).

Now the idea is to define ${\hat{u}}_{\eta }^{\left(q+1\right)}$ such as:

$${\hat{u}}_{\eta }^{\left(q+1\right)}=u_{\eta }^{\left(q\right)}+c_{\eta }^{\left(q+1\right)}$$ (30)

where $c_{\eta }^{\left(q+1\right)}$ can be seen as a degree *q* + 1 correction to the degree *q* approximate solution $u_{\eta }^{\left(q\right)}$, thus the name hierarchical error estimator. The error bound Eq (29) reduces to

$$\|u-u_{\eta }^{\left(q\right)}\|\leq \frac{1}{1-\beta }\left\|c_{\eta }^{\left(q+1\right)}\right\|$$ (31)

In order to construct $\left\|c_{\eta }^{\left(q+1\right)}\right\|$ our method requires an accurate approximation of the gradient (∇*u*) of the solution (denoted $g_{\eta }^{\left(q\right)}$), which we also require to belong to the space of piecewise continuous functions whose restriction to any element ⊤ of $T_{\eta }$ belongs to the space $P_{q}(⊤)$). For this purpose, there exists gradient recovery methods producing accurate approximations of ∇*u*. We will use the one described in [19].

Then $\left\|c_{\eta }^{\left(q+1\right)}\right\|$ can be easily computed using $u_{\eta }^{\left(q\right)}$ and the recovered gradient $g_{\eta }^{\left(q\right)}$. Indeed, to enrich $u_{\eta }^{\left(q\right)}$ in order to get an approximation ${\hat{u}}_{\eta }^{\left(q+1\right)}$ of degree *q* + 1, we need, as in a Taylor expansion, its derivatives of order *q* + 1. Obviously, the (*q* + 1)th derivatives of $u_{\eta }^{\left(q\right)}$ vanish but we can use the *q*th derivatives of the recovered gradient $g_{\eta }^{\left(q\right)}$. This means that the (*q* + 1)th derivative of ${\hat{u}}_{\eta }^{\left(q+1\right)}$ should coincide with the appropriate *q*th derivative of $g_{\eta }^{\left(q\right)}$.

In the general case, a well posed linear system can be built on each element, whose solution completely defines *c*^(*q*+1)^. Now, if we can control the correction *c*^(*q*+1)^, then from Eq (31) we can also control the error and this will serve for the construction of an adapted mesh.

The first objective is to reach a prescribed global target level of error (*e*_Ω_) in *L*^2^-norm.

$$\sum_{⊤\in T_{\eta }}\left|\right|c^{\left(q+1\right)}{\left|\right|}_{0,⊤}^{2}=e_{\Omega }^{2}$$

Since we are modifying the mesh locally, this objective will be reached also locally by imposing on each element

$$\left|\right|c^{\left(q+1\right)}{\left|\right|}_{0,⊤}^{2}=\frac{e_{\Omega }^{2}\;\text{meas}\;\left(⊤\right)}{\;\text{meas}\;(\Omega )}=e_{⊤}^{2}.$$

This way, the error is distributed proportionally to the element area (or volume in 3D) and if this target value can be reached on each element, then the global error *e*_Ω_ will be attained. These local target errors are imposed using edge refinement and node elimination (Fig 1A and 1B). For edge refinement for example, we build for each edge *e* of the mesh the patch $P_{\eta }(e)$ of elements containing *e* (Fig 1A). On that patch, the local target error should be

$$\sum_{⊤\in P_{\eta }\left(e\right)}e_{⊤}^{2}=\frac{e_{\Omega }^{2}\;\text{meas}\;\left(P_{\eta }\left(e\right)\right)}{\;\text{meas}\;(\Omega )}$$ (32)

and the estimated error is

$$\sum_{⊤\in P_{\eta }\left(e\right)}\left|\right|c^{\left(q+1\right)}{\left|\right|}_{0,⊤}^{2}.$$

We then cut that edge by adding a new mid-side node and we reinterpolate the recovered gradient $g_{\eta }^{\left(q\right)}$ at the new node which is necessary for the computation of *c*^(*q*+1)^ on the newly created elements. We then compute the error estimate on the new patch and choose, between the initial patch and the new one, the one for which the estimated error is closest to the target Eq (32). A similar procedure is used for node elimination and more details are given in [11, 12].

We also want to reach the global target error value with a minimum number of nodes. As a second objective, we therefore try to achieve some form of equidistribution of the error by minimizing, as in [20], the *H*^1^-seminorm of the error which is approximated by the quantity:

$${\left(\sum_{⊤\in T_{\eta }}||\nabla c^{\left(q+1\right)}{\left|\right|}_{0,⊤}^{2}\right)}^{1/2}.$$

This minimization is also performed locally but now using edge swapping and node displacement. To move node ***P*** for example, we construct the patch $P_{\eta }(P)$ of elements sharing that node and determine its new position in order to minimize the quantity

$$\sum_{⊤\in P_{\eta }\left(P\right)}||\nabla c^{\left(q+1\right)}{\left|\right|}_{0,⊤}^{2}.$$

A gradient method can be used for the minimization. The node is only allowed to move inside the patch to prevent element inversion. A similar procedure is used for edge swapping. Remarkably, the minimum on the *H*^1^-seminorm of the error cannot be achieved without somehow reorienting and stretching the elements in appropriate directions, therefore leading to anisotropic meshes as shown in [12, 20].

In summary, mesh modification is done by sweeping the nodes (for node elimination and node displacement) and the edges (for edge division and edge swapping) a number of times until the two above objectives are approximately satisfied.

Algorithm 2 summarizes the method used for mesh adaptation.

**Algorithm 2**: Mesh adaptation method

1: Given an initial mesh $M_{0}$.

2: **While** [mesh variations occurs]

3:  Solve to obtain a solution *u*_*i*_ on mesh $M_{i}$.

4:  Compute an estimation of the error Eq (31).

5:  Adapt the mesh $M_{i}$ using local operations to obtain $M_{i+1}$.

  • Reach a prescribed global target level of error in *L*^2^-norm thanks to edge refinement and node elimination.

  • Minimize the *H*^1^-seminorm of the error using node displacement and edge swapping (error equidistribution).

6:  Set *i* = *i*+1.

7: **End while**

Algorithm 2 was performed on an initial mesh generated from COMSOL Multiphysics, a commercial software.

Finally, the method was implemented using the finite element library MEF++ developed at the *Groupe Interdisciplinaire de Recherche en Éléments Finis* (GIREF) (see http://giref.ulaval.ca/mef.html).

## Results

### Gain in accuracy through solution based mesh adaptation

**Fig 2:**
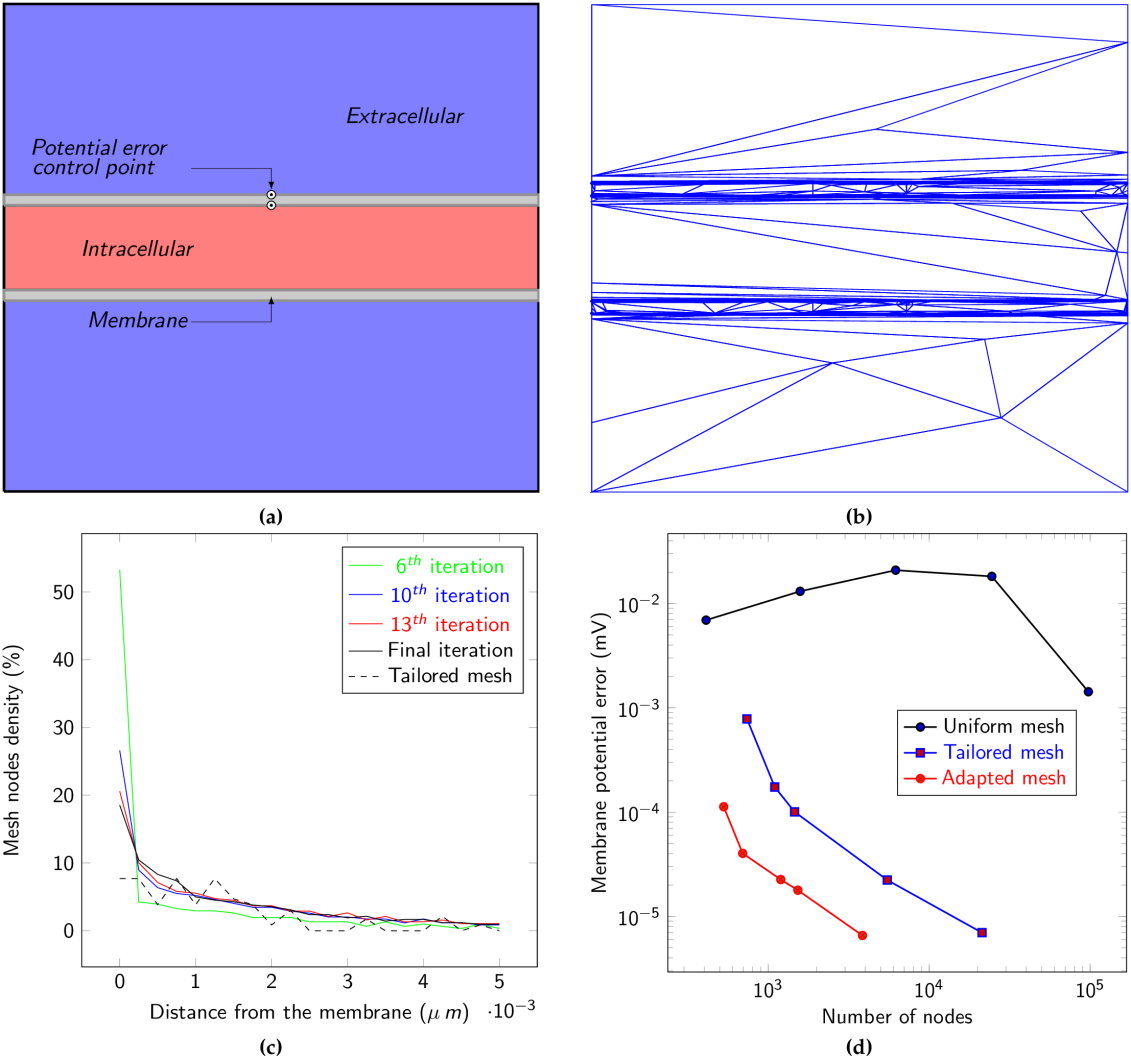
**a)** Schematic of the two dimensional model used to compute the numerical error of the method. The membrane potential was computed by the difference of electric potential at each side membrane (*see the two circles*). **b)** Example of a computational grid obtained with the mesh adaptation method. **c)** Distribution of mesh nodes in the intracellular space as a function of the distance from the membrane. Results obtained for the iterations Algorithm 2 as well as for a tailored mesh. **d)** Membrane potential error (see Eq (37)) on different meshes. The error was computed as a function of the number of computation nodes in the mesh for different strategies: **uniform meshes** with a uniform refinement, **tailored meshes** with increased node density near the interface of the membrane and intra (extra) cellular space and meshes obtained through **mesh adaptation method**.

While the FEM and the finite volume method have been used to describe various problems in neuroscience ([5, 6]), to the best of our knowledge no attempt has been made to quantify the relationship between the numerical error and the mesh size in this context. For the sake of simplicity and in order to be able to compare our results with exact solutions, we first considered the simplest of situations, a two-dimensional geometry with linear membranes and no transmembrane flux (Fig 2A). In this scenario, variations in ionic concentrations and electric potential are localized near the membrane where the electric potential is well described by the Poisson Boltzmann equation

$$\Delta V+\sum_{k=1}^{n^{*}}\frac{z_{k}c_{k}^{0}F}{\varepsilon_{0}\varepsilon_{w}}\exp\left(-\frac{z_{i}FV}{N_{A}k_{B}T}\right)=0$$ (33)

with *k*_*B*_ ≈ 1.38 × 10^−23^ J ⋅ K^−1^ the Boltzmann constant and *N*_*A*_ ≈ 6.022mol^−1^ the Avogadro number. In this context $c_{k}^{0}$ denotes the concentration of ionic specie *k* at infinity (far from the membrane) taken to be equal to its initial value. Linearizing Eq (33) and assuming electroneutrality away from the membrane, one obtains

$$\Delta V=\left(\sum_{k=1}^{n^{*}}\frac{z_{k}^{2}F^{2}c_{k}^{0}}{\varepsilon_{0}\varepsilon_{w}k_{B}TN_{A}}\right)V.$$ (34)

Solving Eq (34) yields the characteristic length of the region in which the electric potential and ionic concentrations vary steeply, i.e. the Debye layer (*λ*_*D*_), according to [13]

$$\lambda_{D}=\sqrt{\frac{\epsilon_{0}\epsilon_{w}k_{B}TN_{A}}{F^{2}\sum_{k=1}^{n^{*}}z_{k}^{2}c_{k}^{0}}}.$$

For the specific values of our problem this gives a Debye length of approximately 1.1 nm.

These observations about the Debye layer led us to formulate three hypotheses: **1)** The mesh adaptation method should concentrate a large proportion the mesh elements within 1 nm of the interfaces between the membrane and the intra-extra cellular spaces, **2)** Mesh node density should decrease in a way that is approximately exponential with distance from the membrane, **3)** Given that variations occur only in the direction perpendicular to the membrane, the adapted mesh should be very anisotropic.

To test these hypotheses, we iteratively applied the mesh adaptation method and solved the PNP equations on the domain (see Algorithm 2). This process led to a very anisotropic mesh with nodes concentrated near the interfaces between membrane and intra-extra cellular spaces (Fig 2B). We created a tailored mesh (see details below) as an alternative to the mesh adaptation method. In Fig 2C we present the nodal distribution, in the intracellular space, as a function of distance from the membrane for the meshes produced by the adaptation process and our tailored mesh. This figure also depicts the evolution of the adaptation process. Here the process was stabilized (i.e. no notable changes in mesh topology observed) after 15 iterations. This clearly demonstrates (Fig 2C) the densification near the membrane in a zone less then 0.25 *nm* from the membrane. Notice the overshoot of the sixth mesh with a peak of nearly 55% of the nodes, which is corrected in the next iterations by redistributing the superfluous nodes in the nearby zone, leading to a mesh with around 20% of the nodes in the first 0.25 *nm* and almost 45% of the nodes in the first nm.

These results can be interpreted as the mesh adaptation algorithm detecting the necessity for the size of some mesh elements to be smaller than 1 nm. However, for a regular mesh to have elements of this size in a 2D geometry of 1 *μ*m^2^ surface area would require 10^6^ elements bearing a prohibitive computational cost. A tailored mesh as proposed by J. Pods et al. [5] is more efficient compared to a uniform mesh. However it requires an a priori knowledge of the solution and in the case of complex geometries, such an approach can become impracticable since properties of the solution may be difficult, if not impossible, to infer in advance. As for the adaptive method, in any cases, it will automatically densify meshes on sensitive areas with respect to the accuracy of the numerical solution. As an indication we added, in (Fig 2C), the nodal distribution for a tailored mesh which is similar to the mesh proposed in [5]. We created this mesh by manually specifying regions of high node density near the membrane and regions of low density elsewhere. The mesh was constructed so that the node density decreases in a geometric manner as a function of the distance from the membrane.

To compare the numerical errors of solutions obtained with different meshing strategies, we took advantage of the easily computed value of the membrane potential

$$V_{\text{mem}}=Q\frac{d_{\text{intra}}d_{\text{mem}}}{2L\varepsilon_{0}\varepsilon_{mem}}$$ (35)

where *L* is the length of the membranes, *d*_intra_ the distance between the two membranes, *d*_mem_ is the width of the membrane (see Fig 2A). The electric permittivity of the membrane is *ε*_*mem*_ and *Q* is the mean electric charge density in the intracellular medium given by

$$Q=\frac{F\sum_{k=1}^{n^{*}}z_{k}c_{k}^{0}}{Ld_{\text{intra}}}.$$ (36)

We compared this exact value to the membrane potential computed by the FEM approach at the center of the membrane. More precisely we computed the absolute difference between the variation of *V*_mem_ and the variation of our approximation *V*_*η*_ on each side of the membrane at control points ${\vec{x}}_{a}=(2,0.434)$ and ${\vec{x}}_{b}=(2,0.534)$ (see Fig 2A):

$$\text{error}=|\left(V_{\text{mem}}\left(\vec{x_{a}}\right)-V_{\text{mem}}\left(\vec{x_{b}}\right)\right)-\left(V_{\eta }\left({\vec{x}}_{a}\right)-V_{\eta }\left({\vec{x}}_{b}\right)\right)|.$$ (37)

The rate (order) of convergence, with respect to the time step and the spatial size of the elements are relatively standard theoretical results (see [21] for instance). The FEM (for continuous coefficients and a regular geometry), would give an error of order *k*^⋆^ with respect to space since we are using degree *k*^⋆^ polynomial approximation in space and of order 2 in time since we are using an order 2 marching scheme (here BDF2). Because we are not fulfilling all the conditions here (our coefficients are discontinuous), and since we are using quadratic polynomial approximation, as presented by Ying and Benzhuo [22] (see also [23]), we “lose” an order of convergence in space leading to a rate of convergence of 1 in space. It should be noted that using a linear approximation in space would lead to a rate of converge in space of 1/2, which strengthens the argument in favour of the use of quadratic interpolation. The approach of mesh adaptation has no effect on the convergence rate since the theoretical results don’t require the discretization parameters to be constant. Of course, in the case of non uniform meshes, error graphs cannot be based on the size of elements which varies throughout the domain, but rather on the number of nodes, which becomes the comparing quantity. Since we have a rate of convergence of 1, the error is expected to be inversely proportional to the number of nodes. We also illustrated the spatial rate of convergence of steady-state solutions (Fig 2D) as we have slopes of 1 for the adapted mesh method as well as for the tailored meshes, indicating that both approaches can be used with confidence in this setting. Notice that the curve for the uniform meshes cannot give us this information as excessively refined meshes would be needed to exhibit a slope of 1. Indeed, the values of membrane potential computed on regular meshes obtained by successive global refinements failed to converge to the exact value one for a number of elements up to 10^5^.

Contrastingly, when we applied the mesh adaptation technique to meshes of different sizes, the error was less 10^−4^ mV for as little as 695 mesh elements (Fig 2D). Using different tailored meshes (obtained by increasing node density in each region) the approximation of the membrane potential converged to the theoretical value with the error reaching 10^−4^ mV for 1458 elements (Fig 2D) therefore needing more then two times the number of nodes used by the adapted mesh for the same order of accuracy.

Since we have a convergence rate of 1 in both cases, the gap in accuracy and in number of nodes will remain, even if we refine the meshes (which corresponds to increasing the number of nodes). Using our initial tailored mesh as a reference, the mesh adaptation method reduces the number of nodes for a given precision by a factor nearly equal to two (Fig 2D). For an accuracy of about 10^−5^, the adaptive method requires approximately 5000 nodes while we would need to refine our initial tailored mesh until it has more than 10000 nodes.

Using tailored meshes with better positioning and density of nodes could produce accuracy arbitrarily close the one obtained using adapted meshes, which are in a sense optimal as discussed above. However, this raises the question of how to determine, with the same efficiency and precision, the zones and the densities of nodes in those regions. Pretending to establish optimal targeted geometrical zones, densities and to control the total number of nodes without tools comparable to those used by the adaptation method (i.e. without a detailed knowledge of the behavior of the error) seems inconceivable.

### Application of mesh adaptation in a model of action potential generation in a node of Ranvier

**Fig 3:**
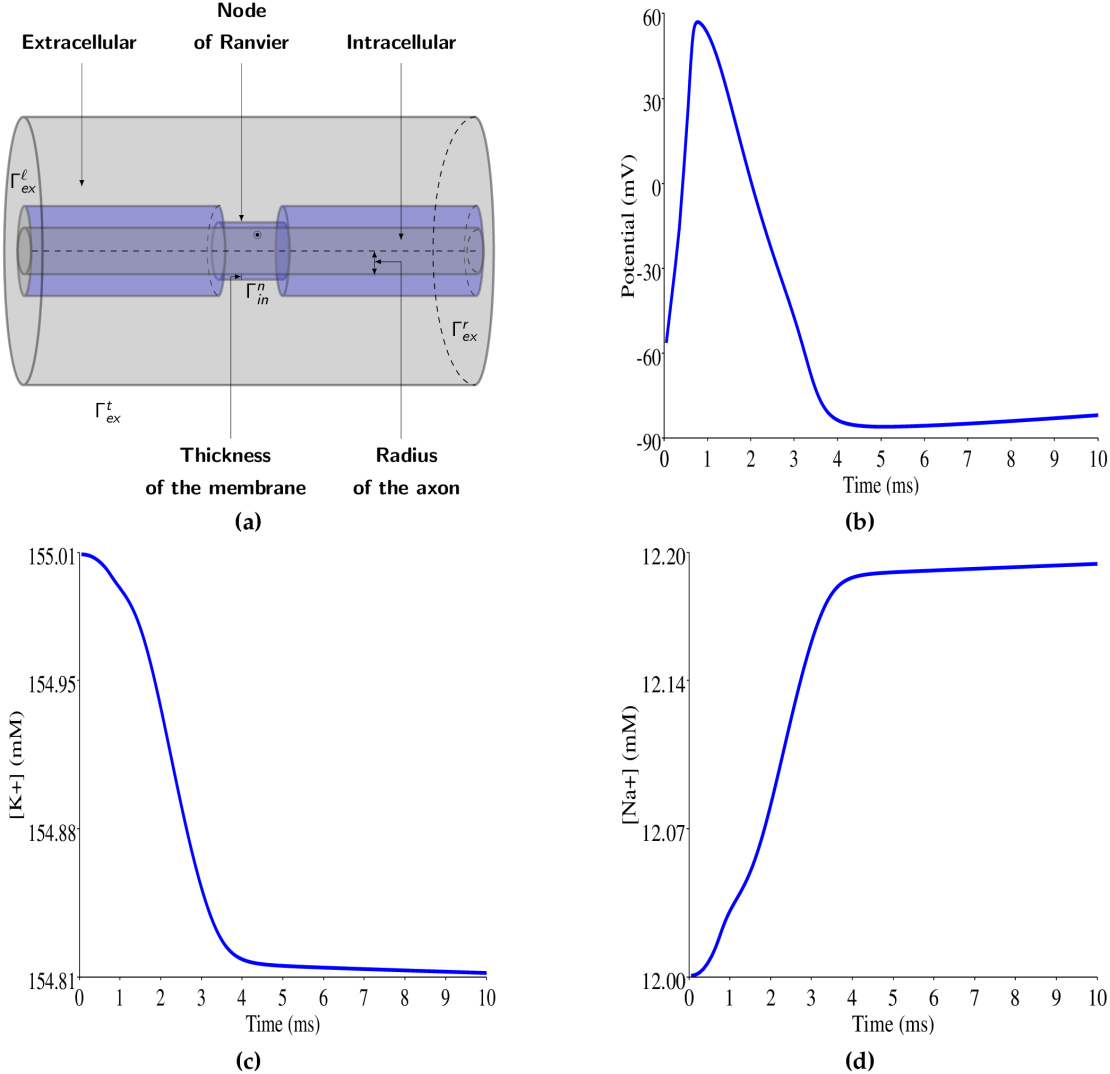
**a)** Schematic of the three dimensional geometry used in the model of a node of Ranvier. **b-d)** Time of electric potential (**b**), potassium concentration (**c**) and sodium concentration (**c**) during an action potential at the spatial point indicated by a circle in **a**.

The advantages of the mesh adaptation method being demonstrated in the simplistic instance of a 2D geometry, we now apply this strategy to the simulation of an action potential in a node of Ranvier. The details of the geometry and most parameters of the model were taken as in [6] while the leak conductance was taken as in [5], see also Fig 3A and Table 2. The action potential was provoked by imposing a [Na^+^] flux in the nodal region during the first 0.5 ms of the simulation. The time course of membrane potential and of intracellular ionic concentrations are shown in Fig 3B–3D and are comparable to results obtained by Lopreore et al. [6].

**Table 2:** Additional parameters for the node of Ranvier.

*d*	0.434*μ*m	Radius of the axon
*D*_*n*_	0.02*μ*m	Thickness of the unmyelinated part of the membrane
*D*_*m*_	0.406*μ*m	Thickness of the myelinated part of the membrane
*L*	4*μ*m	Length of the axon section
*L*_*node*_	0.7*μ*m	Length of node in which currents are applied
$g_{K}^{L}$	0.435mS ⋅ cm^−2^	Conductance density of leak K^+^ channels
$g_{Na}^{L}$	0.065mS ⋅ cm^−2^	Conductance density of leak Na^+^ channels
${\bar{g}}_{K}^{v}$	36mS ⋅ cm^−2^	Conductance density of voltage-gated K^+^ channels
${\bar{g}}_{Na}^{v}$	120mS ⋅ cm^−2^	Conductance density of voltage-gate Na^+^ channels
dur	0.5ms	Duration of the stimulus
Dur	10ms	Duration of the simulation

We adjusted the relative dielectric permittivity of the membrane to obtain an equivalent electric capacitance of 2*μF*/cm^2^ in the node and 0.5*μF*/cm^2^ in the myelinated part of the membrane as in [6]. We made the conversion according to the formula giving electrical capacitance of a cylindrical shell

$$\text{Cap}=\frac{2\varepsilon_{0}\varepsilon_{\text{mem}}\pi L}{\log((D+d)/d)}$$ (38)

where *d* is the radius of the axon and *D* the width of the membrane. This gave the unphysiological value of *ε*_mem_ = 40 (as opposed to the experimentally measured value between 2 and 10 [24]). This might be due to the fact that the width of membrane in the nodal region is larger than the physiological value (20 nm vs ∼ 5–10 nm [25]). The specific values of these parameters have however no impact on the general results of the present paper regarding the benefits of the mesh adaptation method.

**Fig 4:**
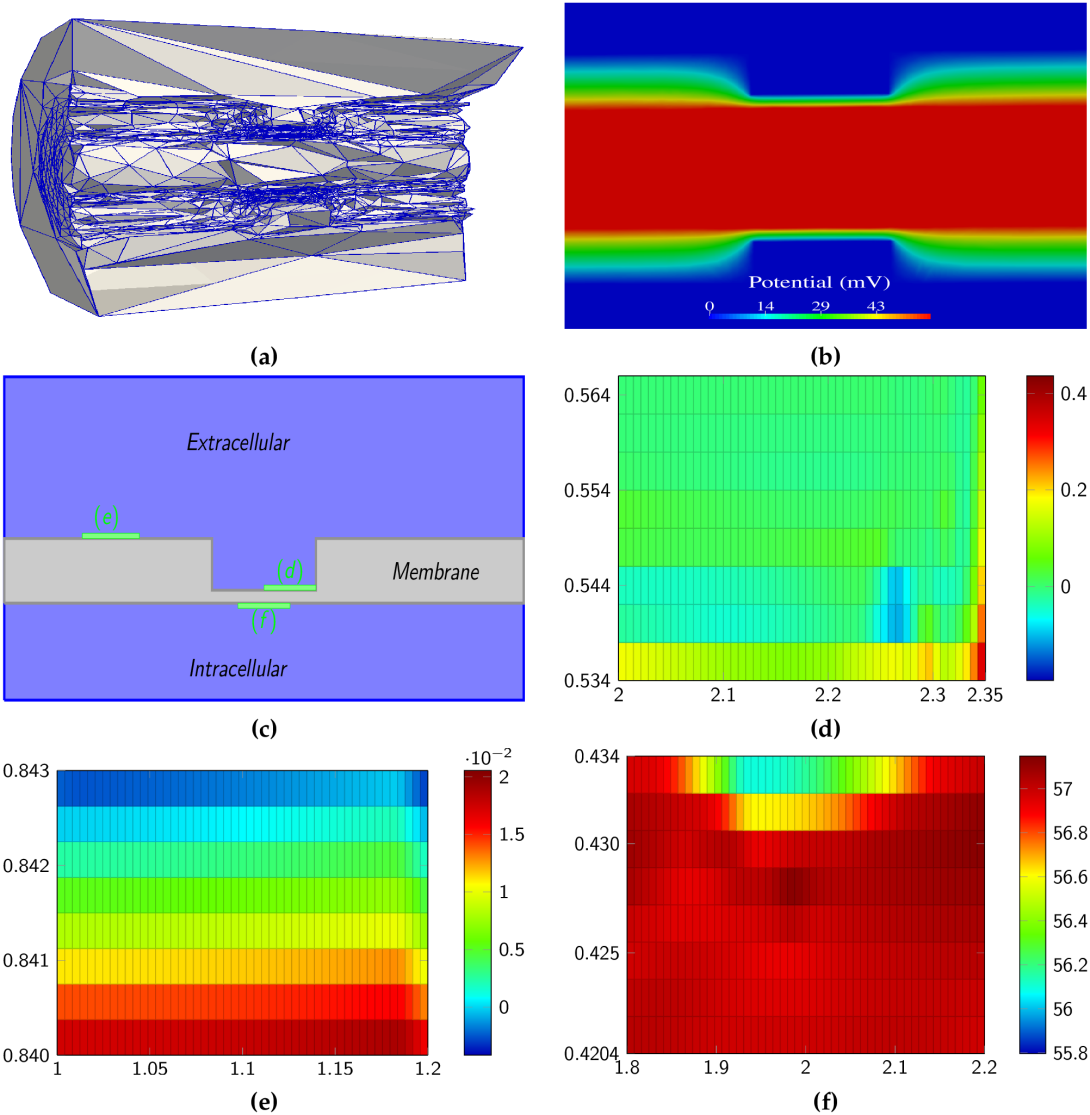
**a)** Illustration of the three dimensional mesh obtained for the description of node of Ranvier by the mesh adaptation method. **b)** Electric potential on the whole domain taken at the peak (most depolarized time point) of the action potential illustrating its non linear distribution on cross-sections of the membrane. **c-e)** Enlarged view of the electric potential in small regions near the membrane illustrating that the model is able to describe the so-called Debye layer.

Highlighting regions of interest of the solution, the mesh adaptation method concentrates mesh elements in and near the unmyelinated part of the membrane while the elements outside of this region are very anisotropic and especially scarce in the extracellular space (Fig 4A). Beyond increasing the accuracy of the results (as described in the previous section), by concentrating the nodes of the grid in regions of interest, adapted meshes can reveal features of the solutions that would otherwise be missed. In particular, the frequent assumption that the electric field is constant in a cross section of the membrane (as is used to derive the GHK flux equation) is no longer valid when the width of the membrane is not uniform as in the present case (Fig 4B). Remark that solving the Poisson equation on complex geometries, especially in the presence of reentering corners, is a problem related to intrinsic mathematical difficulties so it would be difficult to obtain a good approximation of the solution by simpler methods.

Eq (34) suggests that the amplitude of the deviation from neutrality of the electric potential in the Debye layer of the extracellular space is directly proportional to its gradient on the membrane (though the actual picture can be complicated by the presence of transmembrane ionic fluxes). This predicts a larger deviation near the corner between the myelinated and unmyelinated section of the membrane (Fig 4D) than near the middle of the myelinated section (Fig 4E).

## Discussion

Solving the PNP equations with the FEM is a promising approach bound to gain wider use to describe electro-diffusion in neural structures [7]. This method is particularly useful to describe structures with complex geometries such as nodes of Ranvier, dendritic spines or the synaptic cleft and its surrounding [5, 6, 8, 9]. In such contexts, the FEM describes phenomena that other computational approaches fail to capture such as the fine scale distribution of ion concentrations and of the electric potential or the way their variations extend beyond the membrane.

However, discontinuities in dielectric permittivity and ionic diffusion coefficients occurring at the interfaces between the membrane and the intra-extra cellular spaces pose numerical challenges, as the solutions typically exhibit strong variations localized at the vicinity of these interfaces. As demonstrated here, this feature of problems naturally occurring in the field of neuroscience can lead to significant numerical errors if not addressed properly. The efficiency of “tailored meshes” have been demonstrated in the context of an axisymmetric model of propagation of an action potential in an axon with membrane of uniform width [5]. In the present paper, we show that the technique of the mesh adaptation method, which has a proven track record in the field of industrial mathematics, can be applied to describe electrodiffusion by solving the PNP equations on complex three dimensional geometries in neuroscience. In addition of yielding higher performance, this approach has the advantage of not requiring the user to manually specify mesh properties based on possibly incomplete a priori beliefs on the solution.

A potential limitation of the mesh adaptation method is that since this procedure is performed using a solution at a given time, the adapted mesh may no longer be optimal as the features of the solution change over time. In the instance of the propagation of an action potential along an axon for example, an ideal strategy would make the concentration of mesh elements follow the propagation of this action potential since this is where the solution varies most abruptly and where it is most important to describe the details of the solution. A possible way to achieve such a time evolving mesh would be to readapt the calculation grid at several time points of the simulation (at each tenth of millisecond for instance) as we hope to achieve in future research. Another potential limitation of using the FEM to solve the PNP equations is that the use of partial derivative equations implicitly assumes that ionic concentrations are continuous quantities and thus fails to describe the granularity arising from the discrete distribution of ions and of transmembrane proteins such as voltage-gated channels. It has however been demonstrated in [6] that stochastic equations can be coupled with partial derivative equations and capture the variance in the solution due to the stochastic behaviour of channels. It might of interest to go one step further and use a system of stochastic partial derivative equations to account for the probabilistic movement of ions. This could become important for ionic species with low concentration such as Ca^2+^ where the small number of ions in each spatial elements can violate the continuity condition on which the use of diffusion equations is founded.

Finally, the FEM applied to structures with geometry evolving over time leading to mesh deformation is relatively common (for instance, in biology, problems such as the dynamics of visicle shape transformations or mitral valve simulation, in engineering, the extreme deformation and buckling of structures). However the integration of the adaptation techniques for such problems is more recent (see [26]). Such techniques could be applied in the field of computational neurosciences, to describe the birth and growth of dendritic spines [27] or the swelling of cells [28], potentially helping to better understand these important phenomena.

## Conclusion

In the field of neuroscience, experimental data is discovered at an ever increasing pace. Integrating this knowledge in comprehensive models is a great challenge promising to help us better understand the function of neural structures. In order for this to be achieved, the steep computational requirements of modelling micrometric structures with a spatial resolution sufficient to fully explain their function (often required to be in the nanometric range) will have to be addressed. While the raw power of parallel calculations performed on super computers will be required, we believe that many numerical methods developed in the field of industrial mathematics could allow to push back the numerical wall arising in computational neuroscience. As demonstrated in the present paper, the use of the mesh adaptation method greatly reduces the numerical cost of solving the PNP equations to model electro-diffusion while increasing the precision of the solution. We hope that this research will open the door for the modelling of larger structures such as axons spanning of several nodes of Ranvier or multiple axons in a nerve bundle. This could provide insights on how loss of myelin can affect signal propagation [14, 29].

## Supporting Information

S1 Methods Detailed information on the dynamics of voltage-gated channels and on the simulation of the generation of an action potential. (PDF)
