# Supplementary material for: Improved Simulation of Electrodiffusion in the Node of Ranvier by Mesh Adaptation
Source: PLoS One. 2016 Aug 22;11(8):e0161318. doi: 10.1371/journal.pone.0161318 (PMC4993505; doi:10.1371/journal.pone.0161318)
Supplement: S1 Methods — (PDF) [file pone.0161318.s001.pdf]

Supplementary methods

The permeability of the membrane with respect to different ion species determines the flux which is applied. The membrane flux boundary condition f_k defined in the paper is taken from [1]. The transmembrane fluxes for ion species k ([K^+] and [Na^+]) is derived from the Hodgkin-Huxley membrane current $I_k = g_k(V,t)([V] - E_k)$, where E_k is the reversal potential for ion species k given by Nernst equation

$$E_k = \frac{RT}{z_k F} \ln(c_k),$$

where $c_k = \frac{c_k^{out}}{c_k^{in}}$ is the relative change in concentration across the membrane. The membrane flux f_k is then given by

$$f_k = \frac{I_k}{z_k F}. \quad (1)$$

The total conductances for $k = K^+$ or $k = Na^+$ are respectively

$$\begin{aligned} g_K &:= g_K^v + g_K^L, \\ g_{Na} &:= g_{Na}^v + g_{Na}^L. \end{aligned}$$

The leak parts g_K^L and g_{Na}^L, given in Table 2, stand for the leak conductances and the voltage-dependent parts g_K^v and g_{Na}^v are defined by

$$\begin{aligned} g_K^v &:= \overline{g}_K^v n^4, \\ g_{Na}^v &:= \overline{g}_{Na}^v m^3 h, \end{aligned}$$

where $\overline{g}_{Na}^v$ and $\overline{g}_K^v$ stand for the total maximal conductance of the voltage gated Na^+ and K^+ channel, respectively also given in Table 2. The functions m, h and n are time and voltage-dependent gating particles, which take values from the interval $[0, 1]$. The kinetics of this gating particles are defined by the following ordinary differential equations

$$\frac{dn}{dt} = \alpha_n(V)(1-n) + \beta_n(V)n, \quad (2)$$

$$\frac{dm}{dt} = \alpha_m(V)(1-m) + \beta_m(V)m, \quad (3)$$

$$\frac{dh}{dt} = \alpha_h(V)(1-h) + \beta_h(V)h, \quad (4)$$

with the corresponding rate functions

$$\alpha_n(V) := \frac{0.01(V+55)}{1-e^{-0.1(V+55)}}$$
$$\beta_n(V) := 0.125e^{-\frac{V+65}{80}}$$
$$\alpha_m(V) := \frac{0.1(V+40)}{1-e^{-0.1(V+40)}}$$
$$\beta_m(V) := 4e^{-\frac{V+65}{18}}$$
$$\alpha_h(V) := 0.07e^{-0.05(V+65)}$$
$$\beta_h(V) := \frac{1}{1+e^{-0.1(V+35)}}$$

Thus, computing the flux's function (1) requires the resolution of ordinary differential equations (2)-(4). These equations were solved with the same time discretization approach (Gear) that we used to solve the main electrodiffusion equations which is described in the main document.

References

[1] Koch, C. *Biophysics of Computation: Information Processing in Single Neurons*. Oxford University Press, Oxford, UK, 2004.

[2] Pods J, Schonke J et Bastian P. *Electrodiffusion models of neurons and extracellular space using the Poisson-Nernst-Planck equations – Numerical simulation of the intra- and extracellular potential for an axon model*. Biophysical Journal. 2013; **105**: 242-254.
